# The demise of the United Kingdom's forensic science service (FSS): loss of world-leading engine of innovation and development in the forensic sciences

**DOI:** 10.1186/2041-2223-2-4

**Published:** 2011-02-01

**Authors:** Bruce Budowle, Manfred Kayser, Antti Sajantila

**Affiliations:** 1 Institute of Investigative Genetics, Department of Forensic and Investigative Genetics, University of North Texas Health Science Center, Fort Worth, TX, USA; 2 Department of Forensic Molecular Biology, Erasmus MC, University Medical Center Rotterdam, Rotterdam, the Netherlands; 3 Department of Forensic Medicine, Hjelt Institute, University of Helsinki, Helsinki, Finland

## 

Notable advancements in science are often remembered for the contributions they have made. In some cases, the names of the inventors or developers have become icons or hallmarks of scientific disciplines. The key example in forensic genetics is Sir Alec Jeffreys and his invention of DNA fingerprinting for forensic applications. Rarely recognized, however, is the infrastructure that facilitated developments and successes. Recent announcements concerning the pending demise of the Forensic Science Service (FSS) in the United Kingdom prompted us to consider and appreciate the FSS's contributions to the forensic sciences. Thus, we would like to bring to the attention of the greater scientific community the prominent role that the FSS has played in the forensic sciences, in particular its contributions for forensic DNA analysis and the consequences of its demise for national and international forensic science and practice.

For the past quarter century, the forensic sciences have seen substantial advancements and improvements promulgated in part by the advent of the field of forensic DNA analysis. The FSS was the initial player in bringing DNA technology to forensic analysis. In 1985, its scientists had the foresight to partner with Sir Alec Jeffreys to solve the murder of two teenage girls using multilocus restriction fragment length polymorphism technology, marking the beginning of the application of DNA analysis for human identification in criminal investigations. The FSS then continued as one of the few leading institutions worldwide on applying DNA technology to forensic applications, by developing, validating and implementing improved and better capabilities. The technology and the field have evolved and increased substantially since those early days in 1985, and along the way the FSS continued its role as a major contributor to the burgeoning discipline of forensic genetics, by its investigators' publishing the results of their efforts in peer-reviewed journals, making the FSS's knowledge available to the worldwide forensic community. Clearly, the global success story of short tandem repeat profiling in forensic analysis would not have been possible without the leading contributions of the FSS. Various forensic institutions around the world emulated the FSS model, and many forensic scientists worldwide owe their scientific and everyday practice heritage to the FSS.

Thus, we were dismayed to read that the UK government has decided to dismantle the FSS. No longer will there be this organization, that has contributed to the foundations of forensic genetics and other forensic science disciplines. Indeed, we cannot think of any other forensic institution worldwide that has contributed more to the advancement of the forensic sciences than the FSS. It is a tragic state of affairs indeed that the UK government is willing to dismiss its own forensic treasure with negative consequences well beyond the borders of the United Kingdom.

In our opinion, the demise of the FSS is not the fault of its scientists; instead, it is the result of an imposed change in infrastructure. The problem originates from an attempt to invoke a business model for all forensic services, and the failure was predicted by many of us back in the 1990 s, when the UK government decided to privatize the FSS (as a government-owned company). We strongly believe that forensic services for criminal investigations should not be thought of as a business. While budget constraints, do affect which services and how much of them can be provided, a cost-benefit business model may jeopardize the UK government's responsibility to protect and secure members of its society. Consider a bank robbery in which the robber takes £8,000. The investigation, arrest and conviction of the robber could cost £50,000. On a cost-benefit basis, it would be more cost-effective for the government to give the £8,000 back to the bank instead of pursuing the robber. This scenario is hardly imaginable in real life, however, despite its economic advantage. We are being somewhat facetious with our example, but the point to be made here is that there are indeed issues that fall under the responsibility of the government that surpass solely that of a business, and forensic services is one of them. The issue is providing stability, safety and security. A government should commit to protecting its people, and forensic science services are one of those capabilities that should be in its arsenal to fight crime. The FSS should never have been put under a business model.

Certainly, one could argue that other entities in the United Kingdom, such as the company LGC, have a successful forensic service business model and that private industry competition can be beneficial. However, success of LGC is in part owing to the existence of the FSS. Indeed, in the past, the motivation for success at the FSS was based on the fact that it was one of a very few agencies with both development and service functions. Without a doubt, traditionally the FSS provided more innovation and fostered more collaborations than any private concerns did. The dedicated, government forensic effort supported innovation and focused on implementing those developments for the service function. We have the utmost respect for the FSS and the contributions its scientists have made.

As the process to eliminate the FSS is being considered, we urge UK policy makers to pause and reconsider. Instead of dissolution, we urge breathing life back into the FSS to bring it back as a true governmental agency, and thus out of a business model, thereby enabling the FSS to continue being the innovator, collaborator and world leader of the forensic sciences. Alternatively, if the UK government does not heed the concerns, we then urge other countries to review what is happening there and to use it as a model case to avoid the unfortunate loss of public forensic services with all its negative national and international consequences.

Mark Jobling has also commented on the closure of the FSS in his February column, available here http://www.investigativegenetics.com/content/2/1/5.

